# Three new species of the genus *Zodarion* (Araneae, Zodariidae) from China

**DOI:** 10.3897/zookeys.813.29683

**Published:** 2019-01-07

**Authors:** Bao-Shi Zhang, Feng Zhang

**Affiliations:** 1 College of Life Sciences, Hebei Normal University, Shijiazhuang, Hebei 050024, China Hebei Normal University Hebei China; 2 Department of Biochemistry, Baoding University, Baoding, Hebei 071051, China Baoding University Baoding China; 3 The Key Laboratory of Invertebrate Systematics and Application, College of Life Sciences, Hebei University, Baoding, Hebei 071002, China Hebei University Baoding China

**Keywords:** Asia, *italicum* group, *lutipes* group, taxonomy, zodariides

## Abstract

Three new species of the genus *Zodarion* Walckenaer, 1826, from China, are described as *Zodarionapertum***sp. n.** (♂♀, from Xinjiang), *Z.planum***sp. n.** (♂, from Shaanxi), and *Z.ovatum***sp. n.** (♂♀, from Yunnan).

## Introduction

The genus *Zodarion* was established by Walckenaer in 1826, with *Enyonitida* Audouin, 1826 as its type species. Jocqué (1991) synonymized *Argus* Walckenaer, 1842, *Clotho* Walckenaer, 1837, *Enyo* Savigny & Audouin, 1825, *Lucia* C. L. Koch, 1837, and *Metargus* F. O. Pickard-Cambridge, 1902 with *Zodarion*. Marusik and Koponen (2001) transferred seven Central Asian and Chinese *Zodarion* species to *Zodariellum*. However, Jocqué and Henrard (2015) rejected the conclusions of Marusik and Koponen (2001) and transferred sixteen species (including two Chinese species) from *Zodariellum* to *Zodarion.*

Presently, *Zodarion* is the largest genus of the subfamily Zodariinae, and includes 160 species. The genus has been recorded in European, Asian, and North African countries (Jocqué 1991; Bosmans 1994, 1997, 2009; Marusik and Koponen 2001; Pekár et al. 2011; Bosmans et al. 2014; Jocqué and Henrard 2015; Li and Lin 2016; WSC 2018), and each species has a limited distribution. From Asia, 34 species are known. 24 species are only known from females and 14 from males (World Spider Catalog 2018). Species of *Zodarion* are rare in China, with only three documented species: *Zodarionchaoyangense* Zhu & Zhu, 1983, from Liaoning and Hebei provinces; *Z.furcum* Zhu, 1988 from Hebei Province; and *Z.hunanense* Yin, 2012 from Hunan Province (Li and Lin 2016). During the examination of spider collections from China, we recognized three new zodariid species which are described here as *Zodarionapertum* sp. n., *Z.planum* sp. n., and *Z.ovatum* sp. n.

## Materials and methods

All specimens have been preserved in 75% ethanol and were examined, illustrated, and measured under a Tech XTL-II stereomicroscope equipped with an Abbe drawing tube. Photographs were taken with a Leica M205A stereomicroscope fitted with a Leica DFC550 camera and LAS software (ver. 4.6). Carapace length was measured medially from the anterior margin to the rear margin of the carapace. Eye size was measured as the maximum diameter of the lens in dorsal or frontal view. The measurements of legs are shown as total length (femur, patella, tibia, metatarsus, tarsus). Only one paratype was measured. Epigynes were cleared in a warm solution of potassium hydroxide, and then transferred to 75% ethanol for drawing. All measurements are given in millimeters. All specimens studied are deposited in the Museum of Hebei University (**MHBU**), Baoding, China.

The following abbreviations are used in the text and figures legends:

**ALE** anterior lateral eyes;

**AME** anterior median eyes;

**MOQ** median ocular quadrangle;

**PLE** posterior lateral eyes;

**PME** posterior median eyes;

**Z**Zodariidae.

## Taxonomy

### Family Zodariidae Thorell, 1881

#### Genus Zodarion

Taxon classificationAnimaliaAraneaeZodariidae

Thorell, 1887

##### Type species.

*Enyonitida* Audouin, 1826.

Most *Zodarion* species are active at night and hide during the day in self-made retreats. Jocqué (1991) provided a generic diagnosis: the number of spinnerets reduced to two in males and six in females, the dense cover of flattened incised hairs on the tegument, and no more than one dorsal femoral spine. Bosmans (1994, 1997, 2009) revised the European *Zodarion* and classified them into 12 groups. Large AME are widely distributed among the taxa in the femoral organ clade, though species of the *Z.thoni*-group have small AME, which is one of the diagnostic characters of *Zodariellum*. *Z.apertum* and *Z.planum* are close to Central Asian spiders considered by Marusik and Koponen (2001) in *Zodariellum*, all these species have similar RTA and bulb. Therefore, the genus *Zodarion* needs to be carefully examined and revised in the future.

#### Zodarion apertum sp. n.

Taxon classificationAnimaliaAraneaeZodariidae

http://zoobank.org/029D1AF1-EEBA-410B-91F1-A92C12B7E6E3

Figures 1 , 2 , 3

##### Type material.

**Holotype** ♂ (Z-Xinjiang-200905-11), Luntai County (41°22'N, 84°11'E), Xinjiang Uygur Autonomous Region, China, 27 May 2009, Dong Sun leg. **Paratype**: 1 ♀ (Z-Xinjiang-200905-12), same data as holotype.

##### Diagnosis.

The male of *Z.apertum* sp. n. is very similar to that of *Z.mongolicum* (Marusik & Koponen, 2001) in having a fold on the apical tegular apophysis, a relatively wider bulb, and the wide and flat apical part of the retrolateral tibial apophysis enlarged. However, it can be distinguished from *Z.mongolicum* by the posteriorly downward direction of the embolic base (down-retrolaterally in *Z.mongolicum*), the retrolateral tibial apophysis with aclinal apical margin in ventral view (flat in *Z.mongolicum*), and the posterior tip of the conductor being at the 6-o’clock-position (5-o’clock-position in *Z.mongolicum*) (Figs 2A–C, 3A–C). The female of this new species resembles that of *Z.zebra* Charitonov, 1946, *Z.spasskyi* Charitonov, 1946, and *Z.proszynskii* Nenilin & Fet, 1985 in having a straight margin of the pocket and closer apices of the two spermathecae, though the spiracles of the spermathecae are smaller than in the latter three species (Figs 2D, E, 3D, E).

##### Etymology.

The specific name is from the Latin *apertum*, in reference to the uncovered terminal of the retrolateral tibial apophysis; adjective.

##### Description.

**Figure 1.**
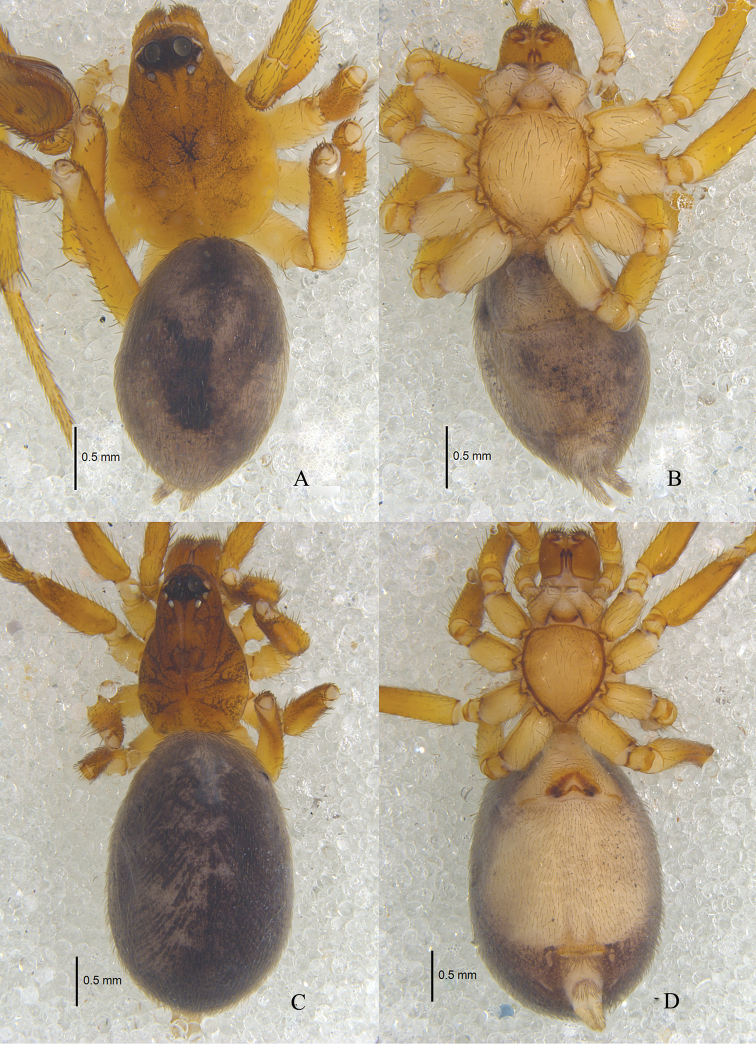
*Zodarionapertum* sp. n., habitus (**A, C** dorsal view **B, D** ventral view) **A, B** Male holotype **C, D** Female paratype.

Male (holotype): total length 3.65; carapace 1.79 long, 1.37 wide; opisthosoma 2.02 long, 1.33 wide. Carapace (Fig. 1A) declining, yellow-brown, furnished with black net-like stripes, flat thorax, and smooth tegument. Longitudinal fovea black. Clypeus 0.16 high, yellow brown. Anterior eye row slightly procurved, posterior eye row strongly procurved in dorsal view. Ocular area black. Eye sizes and interdistances: AME 0.18, ALE 0.12, PME 0.08, PLE 0.10, AME–AME 0.08, AME–ALE 0.01, ALE–ALE 0.55, AME–PME 0.07, PME–PME 0.24, PME–PLE 0.04, PLE–PLE 0.52, ALE–PLE 0.03. MOQ 0.32 long, anterior width 0.41, posterior width 0.40. Mouthparts (Fig. 1B): chelicerae yellow-brown, with two anterior and one posterior teeth on margins of fang furrows, terminal part with row of black hairs; endites yellowish, apices paler and provided with dense black scopula; labium triangular, 0.23 long, 0.29 wide, yellow-brown, apices paler. Sternum (Fig. 1B) 0.90 long, 1.04 wide, yellowish, lateral margin dark, provided with sparse black setae, its lateral margin with inter- and intra-coxal triangles. Legs (Fig. 1A, B) yellow-brown. Leg measurements: leg I 5.39 (1.05 + 0.51 + 1.47 + 1.66 + 0.70), II 4.47 (0.90 + 0.46 + 1.54 + 1.00 + 0.57), III 5.85 (1.68 + 0.48 + 1.53 + 1.52 + 0.64), IV 6.24 (1.57+ 0.58 + 1.31 + 1.70 + 1.08). Opisthosoma covered with pale short hairs. Dorsum of opisthosoma gray, covered with large irregular black patches; venter gray, with small black patches. Spinnerets gray (Fig. 1A, B).

**Figure 2.**
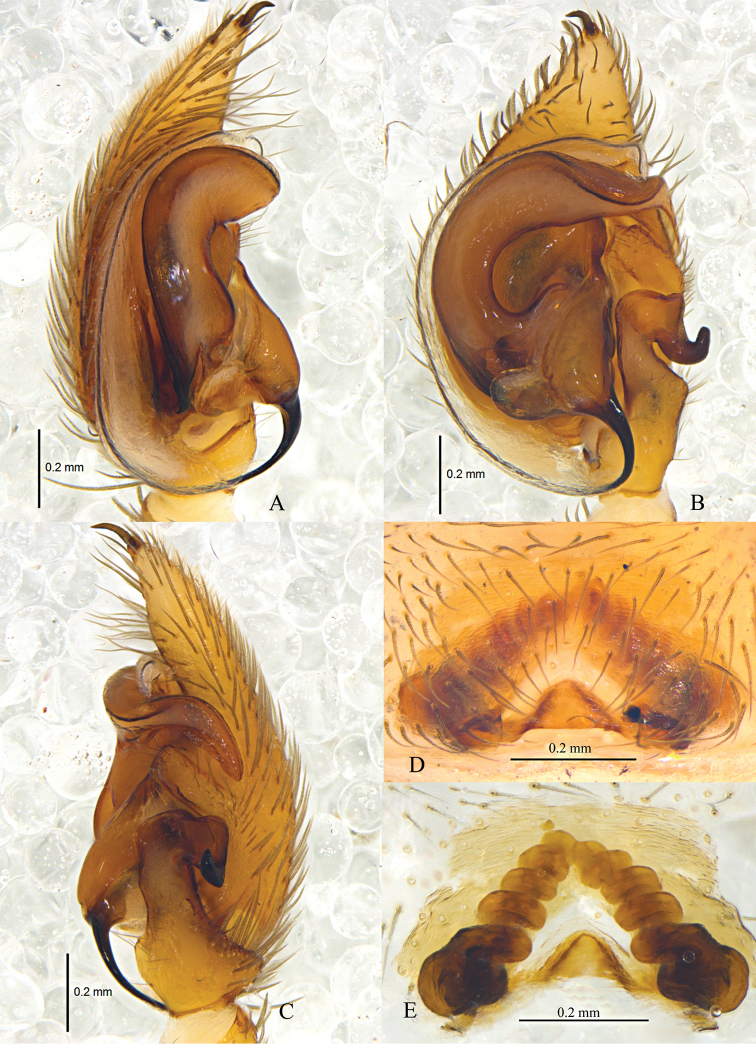
*Zodarionapertum* sp. n., male holotype left palp (**A–C**) and female paratype (**D, E**) **A** Prolateral view **B** Ventral view **C** Retrolateral view **D** Epigynum, ventral view **E** Vulva, dorsal view.

**Figure 3.**
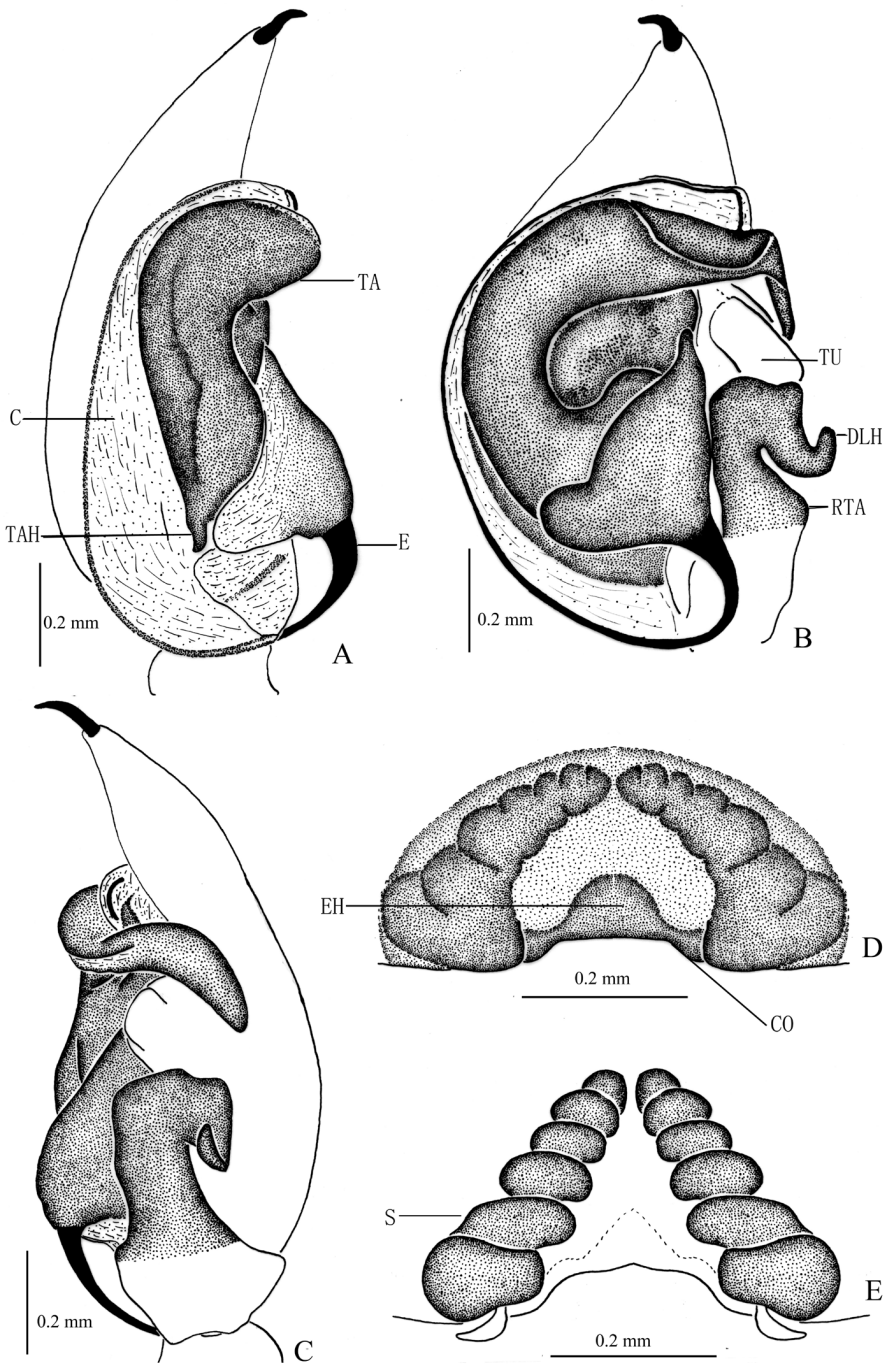
*Zodarionapertum* sp. n., male holotype (**A–C**) and female paratype (**D, E**) **A–C** Left male palp (**A** prolateral view **B** ventral view **C** retrolateral view) **D** Epigynum, ventral view **E** Vulva, dorsal view. Abbreviations: C, conductor; DLH, dorsolateral hook; E, embolus; EH, epigynal hood; RTA, retrolateral tibial apophysis; S, spermatheca; TA, tegular apophysis; TAH, tegular apophysis hook; TU, tutaculum.

Palp (Figs 2A–C, 3A–C). Coxae of palps white, other sections yellow; length to width ratio of femur 3.0, length to width ratio of patella 1.2; retrolateral tibial apophysis enlarged, about 3 times the tibial length, apical part wide and flat, apical margin aclinal, with thin hook-shaped dorsolateral terminal; cymbium with terminal spine, tutaculum obvious; tegular apophysis wide and strong, apical part with a fold, retrolaterally with long and beak shaped extension, tegular apophysis hook S-shaped in prolateral view, extends to basal embolus; membranous conductor long, lamellate and running almost along whole course of the embolus; base of embolus almost triangular.

**Female.** Total length 4.65: carapace 1.73 long, 1.12 wide; opisthosoma 2.96 long, 1.92 wide. Carapace yellow-brown. Clypeal height 0.21. Eye diameters: AME 0.19, ALE 0.11, PME 0.08, PLE 0.08. Eye sizes and interdistances: AME–AME 0.07, AME–ALE 0.02, ALE–ALE 0.51, AME–PME 0.08, PME–PME 0.22, PME–PLE 0.04, PLE–PLE 0.48, ALE–PLE 0.04. MOQ 0.32 long, front width 0.36, back width 0.33. Mouthparts (Fig. 1D): labium 0.23 long, 0.31 wide. Sternum 0.83 long, 0.97 wide. Leg measurements: I 4.78 (1.28 + 0.55+ 1.06 + 1.30 + 0.59), II 4.58 (1.30 + 0.42 + 0.93 + 1.23 + 0.70), III 4.73 (1.24 + 0.45 + 1.02 + 1.28 + 0.74), IV 6.14 (1.62 + 0.53 + 1.39 + 1.62 + 0.98). Dorsum of opisthosoma black; venter white. Other colors as in male (Fig. 1C, D).

Epigyne (Figs 2D, E, 3D, E). Posterior part with central hood, which is bathingcap-shaped, 0.6 long, 0.13 wide, posteromedian margin straight, copulatory openings situated in posterior part of epigyne; long and spiraled spermathecae visible through integument, apices of two spermathecae converging and touching each other, base of two spermathecae distant (about 4 times the spermathecal diameter), brown spermathecae with 5 coils.

##### Distribution.

China (Xinjiang).

#### Zodarion planum sp. n.

Taxon classificationAnimaliaAraneaeZodariidae

http://zoobank.org/CAB3D10A-A662-4AEC-8829-35D9157EFDB3

Figures 4 , 5

##### Type material.

**Holotype** ♂ (Z-Shaanxi-198606-22), Baoji City (34°22'N, 107°09'E), Shaanxi Province, China, 22 June 1986, Mingsheng Zhu leg.

##### Diagnosis.

**Figure 4.**
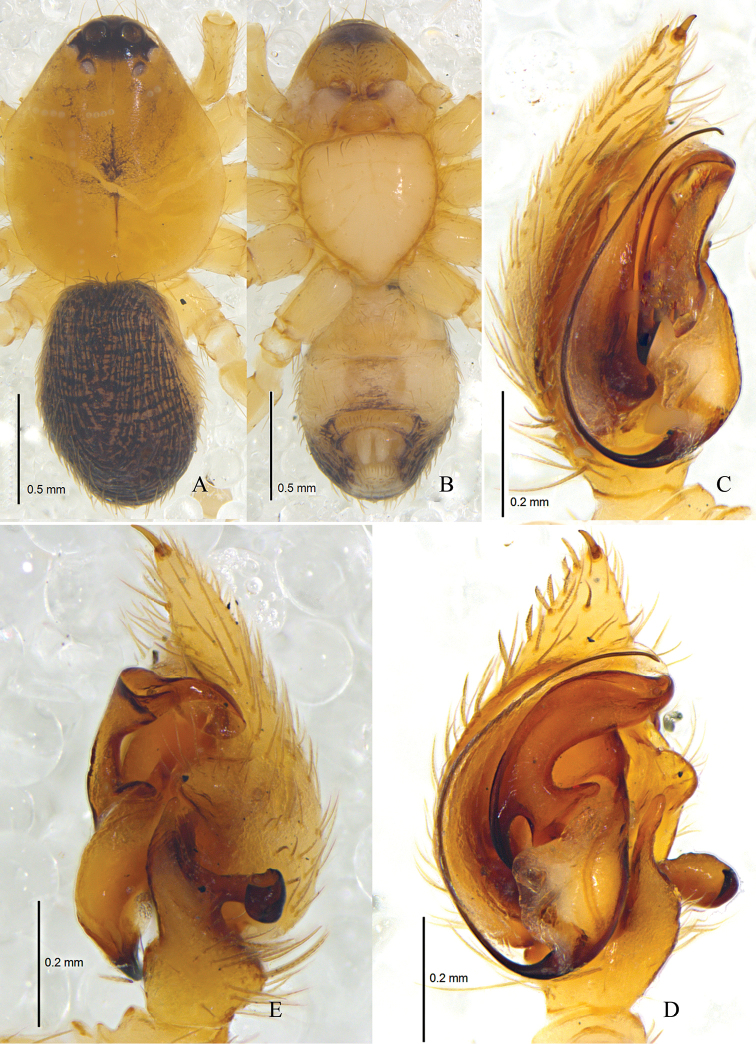
*Zodarionplanum* sp. n., male holotype (**A–E**) **A, B** Habitus (**A** dorsal view **B** ventral view) **C–E** Left male palp (**C** prolateral view **D** ventral view **E** retrolateral view).

**Figure 5.**
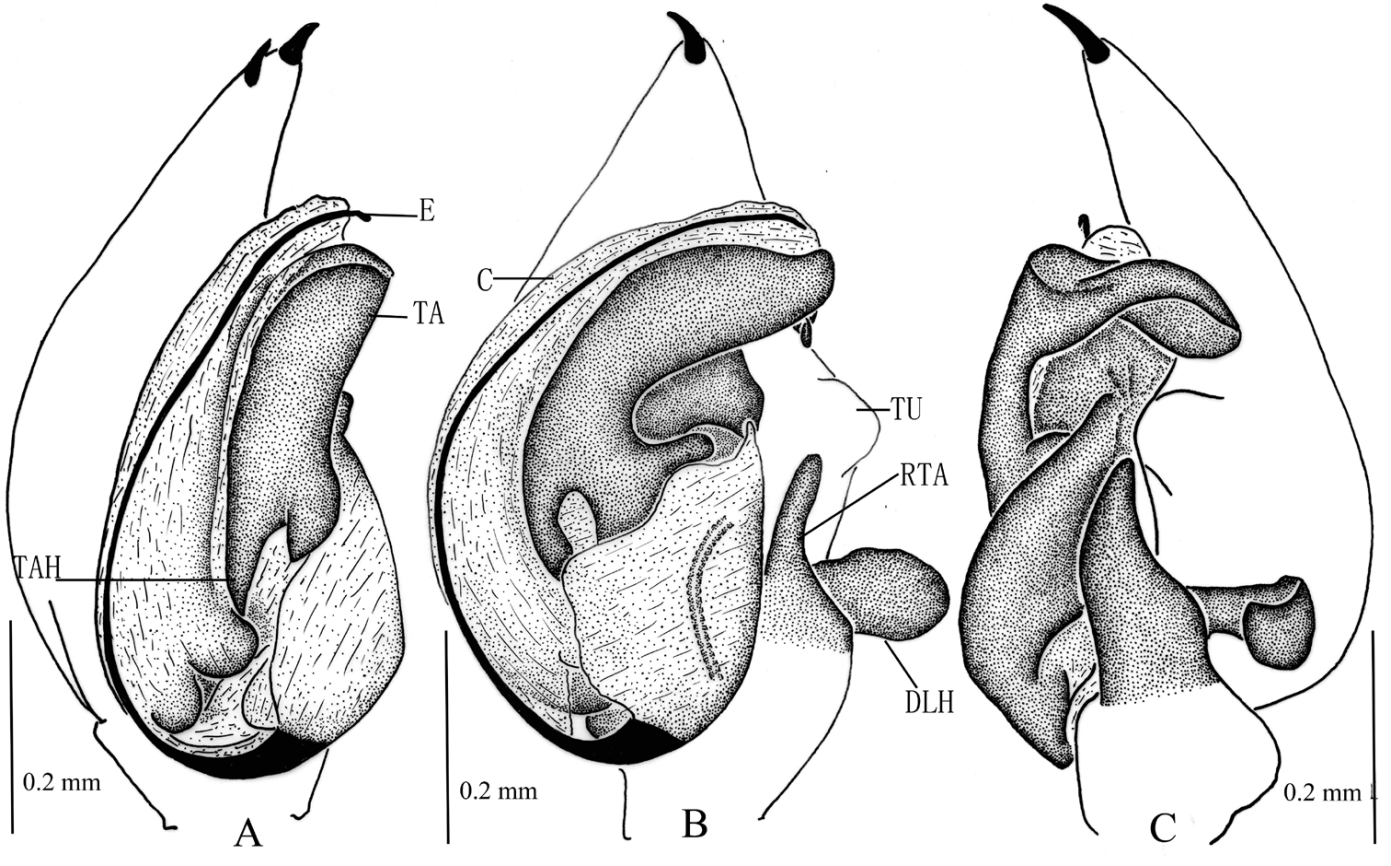
*Zodarionplanum* sp. n. **A–C** Left male palp of male holotype (**A** prolateral view **B** ventral view **C** retrolateral view). Abbreviations: C, conductor; DLH, dorsolateral hook; E, embolus; RTA, retrolateral tibial apophysis; TA, tegular apophysis; TAH, tegular apophysis hook; TU, tutaculum.

The male of *Z.planum* is very similar to those of *Z.sytchevskajae* Nenilin & Fet, 1985, *Z.chaoyangense* Zhu & Zhu, 1983, and *Z.furcum* Zhu, 1988, as all have dorsolateral processes extending from the middle part of the retrolateral tibial apophysis, though *Z.planum* can be distinguished from the others by the wide and fluent margins of the dorsolateral process (obviously curving in the other three species) (Figs 4C–E, 5A–C).

##### Etymology.

The specific name is from the Latin *planum*, in reference to the dorsolateral hook-shaped process of the retrolateral tibial apophysis; adjective.

##### Description.

Male (holotype): total length 2.22; carapace 1.20 long, 1.00 wide; opisthosoma 1.02 long, 0.74 wide. Carapace (Fig. 4A) declining, longer than wide, yellow-brown, furnished with inconspicuous black netlike stripes. Clypeus 0.16 high, yellow-brown. Anterior eye row slightly procurved, posterior eye row strongly procurved in dorsal view. Ocular area black. Eye sizes and interdistances: AME 0.13, ALE 0.08, PME 0.07, PLE 0.07, AME–AME 0.04, AME–ALE 0.06, ALE–ALE 0.42, AME–PME 0.08, PME–PME 0.16, PME–PLE 0.02, PLE–PLE 0.42, ALE–PLE 0.02. MOQ 0.26 long, anterior width 0.28, posterior width 0.29. Mouthparts (Fig. 4B): chelicerae yellow-brown, with two anterior and one posterior teeth on margins of fang furrows, terminal part with row of black scopulae, fangs short; endites yellowish, apices paler and provided with dense black scopula; labium triangular, 0.16 long, 0.25 wide, yellow-brown. Sternum (Fig. 4B) 0.70 long, 0.64 wide, white, lateral margin dark, provided with sparse black setae, its lateral margin with inter- and intra-coxal triangles. Legs (Fig. 4A, B) yellow-brown; femora with dorsal spines. Leg measurements: leg I 5.32 (0.98 + 0.51 + 1.47 + 1.66 + 0.70), II 2.78 (0.78 + 0.38 + 0.65 + 0.59 + 0.38), III 4.41 (0.92 + 0.38 + 1.54 + 1.00 + 0.57), IV 5.79 (1.12+ 0.58 + 1.31 + 1.70 + 1.08). Opisthosoma (Fig. 4A, B) covered with black short setae. Dorsum of opisthosoma black; venter white, median part with wide dark gray band. Spinnerets (Fig. 4B) white, laterally with blackish patches.

Palp (Figs 4C, D, 5A–C). Coxae of palps white, other sections yellow; length to width ratio of femur 2.7, length to width ratio of patella 1.2; retrolateral tibial apophysis about 2.5 times the tibial length, thin apex finger-shaped, dorsolateral hook-shaped apophysis long and flat; cymbium with terminal spine, tutaculum obvious; tegular apophysis of moderate size, retrolaterally with flat and wide extension, tegular apophysis hook nearly straight in prolateral view; membranous conductor long, lamellate, and running almost along whole course of embolus; basal embolus almost triangular.

Female unknown.

##### Distribution.

China (Shaanxi).

##### Remarks.

The two new species *Z.apertum* sp. n. and *Z.planum* sp. n., together with most known East Asian and Central Asian species of the *Zodarion* (i.e. *Z.asiaticum* Tyschchenko, 1970, *Z.bekuzini* Nenilin, 1985, *Z.chaoyangense* Zhu & Zhu, 1983, *Z.continentale* Andreeva & Tyschchenko, 1968, *Z.furcum* Zhu, 1988, *Z.mongolicum* (Marusik & Koponen, 2001), *Z.nenilini* Eskov, 1995 (also distributed in European area and Urals of Russia), *Z.proszynskii* Nenilin & Fet, 1985, *Z.schmidti* (Marusik & Koponen, 2001), *Z.spasskyi* Charitonov, 1946, *Z.hunanense* Yin, 2012, *Z.sytchevskajae* Nenilin & Fet, 1985, *Z.volgouralensis* (Ponomarev, 2007) (also distributed in Astrakhan of Russia), and *Z.zebra* Charitonov, 1946) appear to comprise an undescribed group with the following common characters: the long and thin embolus rising at the prolateral or basal part of the tegulum; tegular apophysis wide and strong, with a downwardly-directed hook; a modified apical portion of the retrolateral tibial apophysis turning dorsally; cymbium with tutaculum; epigyne with incised posteromedian margin and median hood; apical parts of spiraled spermathecae converging. They are different from the *lutipes* group (Bosmans 2009) in having a long conductor, the tutaculum of the cymbium, and the converging apex of the spermathecae.

*Zodarionhunanense* was described based only on a female specimen from Hunan province of China. The possibility exists that *Z.planum* sp. n. is conspecific with *Z.hunanense*.

#### Zodarion ovatum sp. n.

Taxon classificationAnimaliaAraneaeZodariidae

http://zoobank.org/4C6933CB-C3BA-43C1-A952-05BED360B6B2

Figures 6 , 7 , 8

##### Type material.

**Holotype** ♂ (Z-Yunnan-200505-18), Yuanmou County (25°51'N, 101°45'E), Yunnan Province, China, 26 May 2005, collector unknown. **Paratypes**: 71 ♂ (Z-Yunnan-200505-19 - Z- Yunnan-200505-89) and 33 ♀ (Z- Yunnan-200505-90 - Yunnan-200505-122), same data as holotype.

##### Diagnosis.

The males of *Z.ovatum* sp. n. are similar to those of *Zodarionnitidum* (Audouin, 1826), *Z.christae* Bosmans, 2009, *Z.deltshevi* Bosmans, 2009, and *Z.samos* Bosmans, 2009 because of the flagelliform embolus rising at the basal part of the tegulum, the small tegular apophysis and the retrolateral tibial apophysis terminally pointed in ventral view, though it can be distinguished from the others by the small oval base of the embolus (triangular in the other three species), and the lack of a gland in the base of the cymbium (present in the other three species). The females of *Z.ovatum* sp. n. are similar to female *Z.soror* (Simon, 1873) in having swollen copulatory ducts, and also to *Z.ludibundum* Simon, 1914 and *Z.nigriceps* (Simon, 1873) by the oblique lateral margines of epigyne, but the copulatory ducts of *Z.ovatum* sp. n. are longitudinally arranged (Figs 7A–E, 8A–E) rather than oblique in the three other species.

##### Etymology.

The specific name is from the Latin *ovatum*, in reference to the oval shape of the swollen copulatory ducts; adjective.

##### Description.

**Figure 6.**
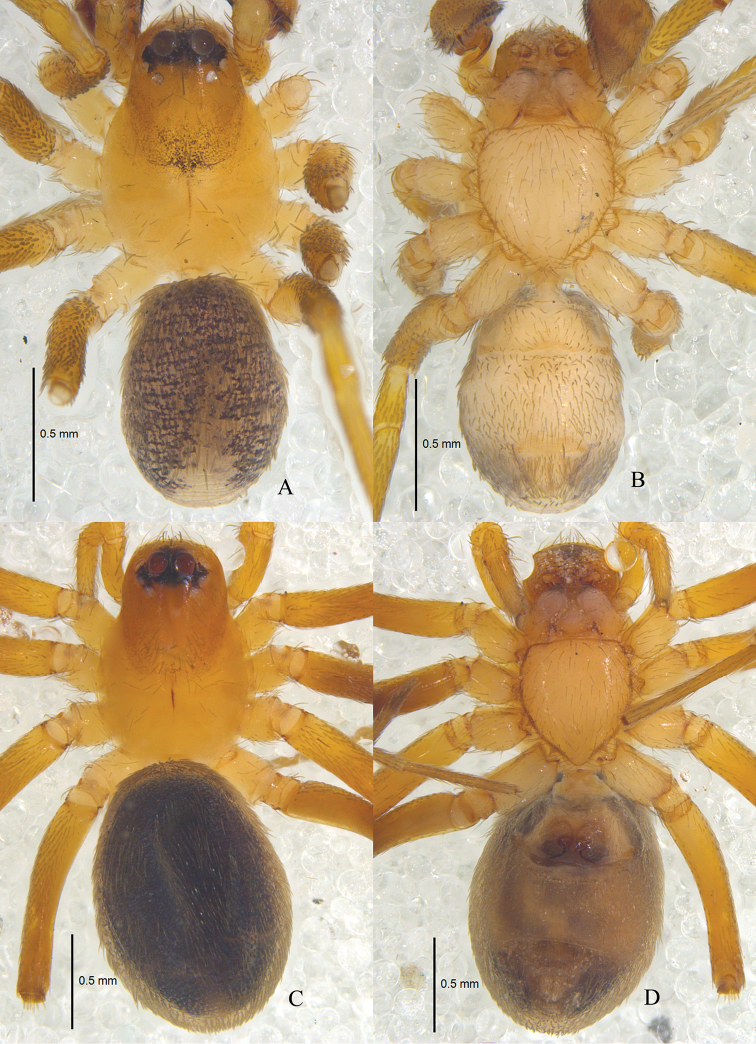
*Zodarionovatum* sp. n., male holotype (**A, B**) and female paratype (**C, D**) Habitus (**A, C** dorsal view **B, D** ventral view).

Male (holotype): total length 1.85; carapace 0.99 long, 0.68 wide; opisthosoma 0.88 long, 0.64 wide. Carapace (Fig. 6A) longer than wide, yellow-brown, median part with faint black patch in front of black fovea, thorax flat, tegument smooth and shiny. Clypeus 0.09 high, yellow-brown. Anterior eye row slightly procurved, posterior eye row strongly procurved in dorsal view. Ocular area black. Eye sizes and interdistances: AME 0.11, ALE 0.03, PME 0.04, PLE 0.04, AME–AME 0.05, AME–ALE 0.03, ALE–ALE 0.33, AME–PME 0.07, PME–PME 0.16, PME–PLE 0.03, PLE–PLE 0.33, ALE–PLE 0.01. MOQ 0.21 long, anterior width 0.24, posterior width 0.25. Mouthparts (Fig. 6B): chelicerae yellow-brown, with two anterior and one posterior teeth on margins of fang furrows, terminal part with row of black scopulae, fangs short; endites yellow, apices paler and provided with dense black scopula; labium triangular, 0.11 long, 0.18 wide, yellow-brown, median part with semi-orbicular brown band. Sternum (Fig. 6B) 0.52 long, 0.55 wide, white, lateral margin dark, provided with sparse black setae, its lateral margin with inter- and intra-coxal triangles. Legs (Fig. 6A, B) brown. Leg measurements: leg I 2.83 (0.75 + 0.26 + 0.59 + 0.78 + 0.45), II 2.54 (0.62 + 0.24 + 0.45 + 0.79 + 0.44), III 2.62 (0.71 + 0.20 + 0.46 + 0.80 + 0.45), IV 3.56 (0.79 + 0.31 + 0.80 + 1.01 + 0.65). Opisthosoma (Fig. 6A, B) oval, covered with black short setae. Dorsum of opisthosoma gray, covered by many dark patches; venter white. Spinnerets (Fig. 6B) pale yellow.

**Figure 7.**
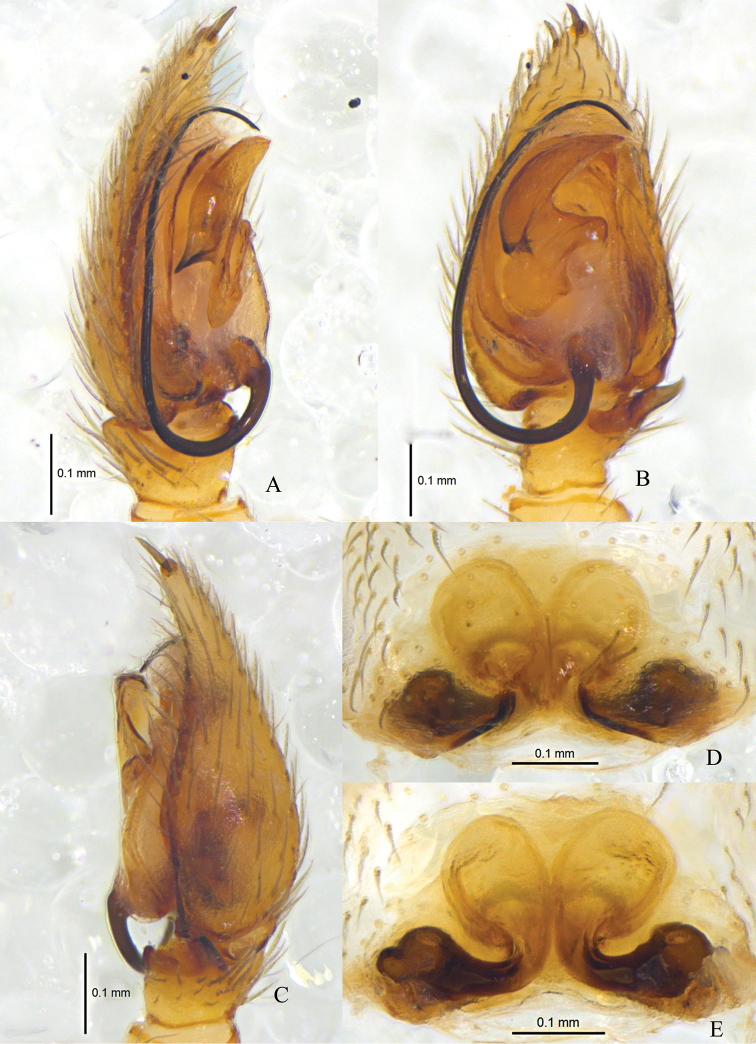
*Zodarionovatum* sp. n., male holotype left palp (**A–C**) and female paratype (**D, E**) **A** Prolateral view **B** Ventral view **C** Retrolateral view **D** Epigynum, ventral view **E** Vulva, dorsal view.

**Figure 8.**
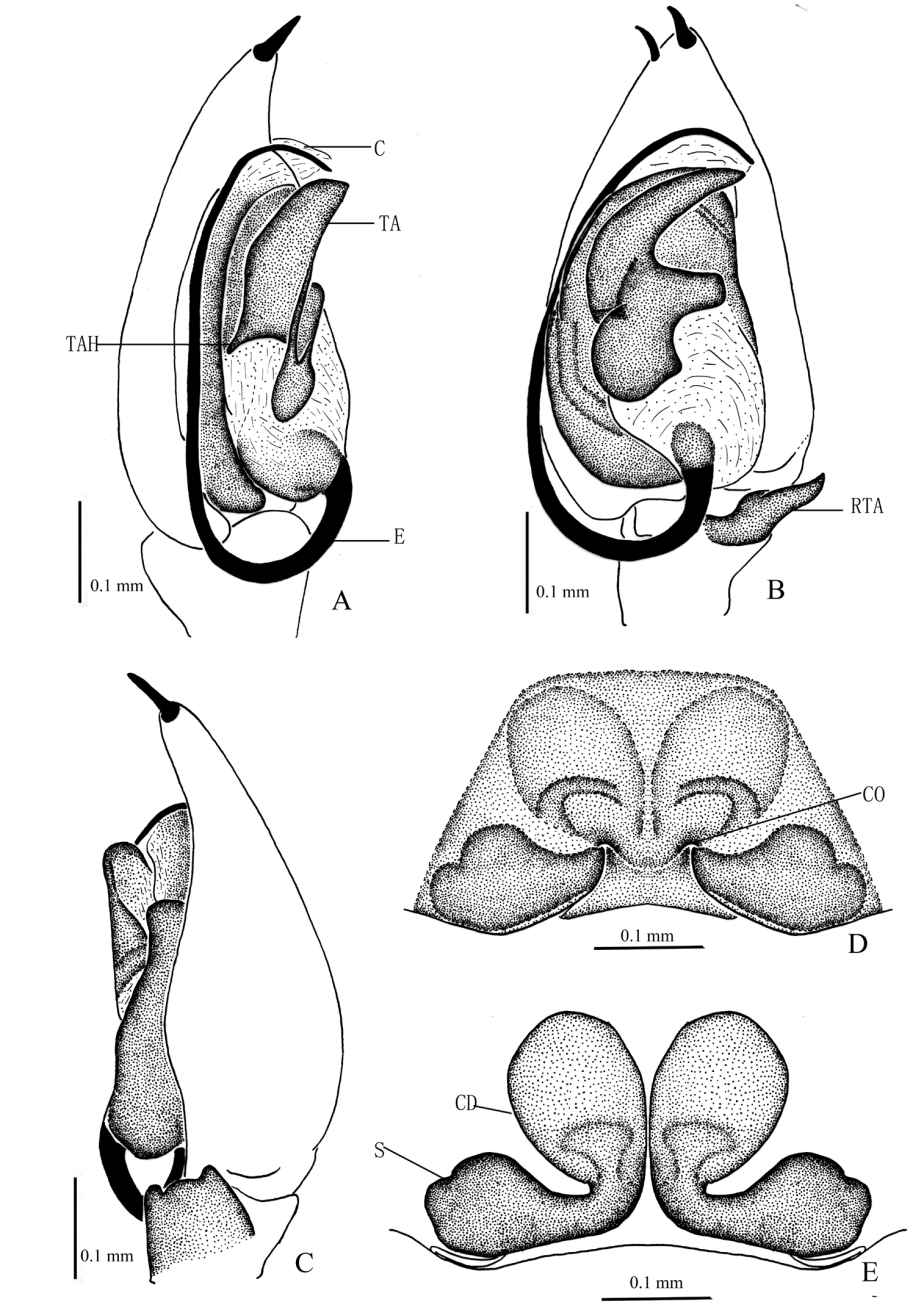
*Zodarionovatum* sp. n., male holotype (**A–C**) and female paratype (**D, E**) **A–C** Left male palp (**A** prolateral view **B** ventral view **C** retrolateral view) **D** Epigynum, ventral view **E** Vulva, dorsal view. Abbreviations: C, conductor; CD, copulatory ducts; CO, copulatory opening; E, embolus; RTA, retrolateral tibial apophysis; S, spermatheca; TA, tegular apophysis; TAH, tegular apophysis hook.

Palp (Figs 7A–C, 8A–C). Palps yellow brown; length to width ratio of femur 2.5, length to width ratio of patella 1.3; retrolateral tibial apophysis as long as tibia, thin and slightly curved in ventral view, but wide in retrolateral view, without dorsolateral process; tegular apophysis large and complex, tip turning gradually tapering, hook of tegular apophysis pointed posteriorly in prolateral view; membranous conductor short; base of embolus small and oval, connected to tegulum via white membrane.

**Female.** One of the specimens (Z-Laos-11–25) measured: total length 2.65; carapace 1.20 long, 0.84 wide; opisthosoma 1.48 long, 1.05 wide. Carapace yellow-brown. Clypeal height 0.09. Eye sizes and interdistances: AME 0.13, ALE 0.09, PME 0.05, PLE 0.08, AME–AME 0.04, AME–ALE 0.03, ALE–ALE 0.41, AME–PME 0.06, PME–PME 0.21, PME–PLE 0.02, PLE–PLE 0.38, ALE–PLE 0.02. MOQ 0.60 long, front width 0.70, back width 0.26. Mouthparts (Figure 1D): labium 0.17 long, 0.21 wide. Sternum 0.83 long, 0.97 wide. Leg measurements: I 2.66 (0.71 + 0.22 + 0.59 + 0.50 + 0.64), II 2.37 (0.81 + 0.17 + 0.47 + 0.64 + 0.28), III 2.54 (0.72 + 0.23+ 0.34 + 0.79+ 0.46), IV 3.79 (1.07 + 0.27 + 0.63 + 1.18 + 0.64). Dorsum of opisthosoma black; venter gray-brown. Other coloration as in male (Fig. 6C, D).

Epigyne with two oblique chitinous sutures, copulatory openings situated almost in the central part of epigyne; anterior part of copulatory ducts swollen, visible through integument; spermathecae small, situated posteriorly and well separated (Figs 7D, E, 8D, E).

##### Distribution.

China (Yunnan).

##### Remarks.

The males of species *Z.ovatum* sp. n. belong to the *lutipes* group with their long embolus rising at the posterior part of the tegulum; tibial apophysis short, robust and without lateral process. The females of *Z.ovatum* sp. n. resemble the species of the *italicum* group (Bosmans 1997) with their parallel or converging chitinous sutures on the epigyne and swollen copulatory ducts.

#### Zodarion chaoyangense

Taxon classificationAnimaliaAraneaeZodariidae

Zhu & Zhu, 1983

Figures 9 , 10

Zodarion chaoyangensis Zhu and Zhu 1983: 137, fig. a–h; Song et al. 1999: 431, fig. 257P, Q, 258A; Song et al. 2001: 327, fig. 210A–D; Jocqué and Henrard 2015: 21.
Zodariellum chaoyangense : Marusik and Koponen 2001: 41.

##### Material examined.

1♂ and 1♀, Zanhuang County (37°65'N, 114°35'E), Hebei Province, China, time and collector unknown.

##### Description.

See Zhu and Zhu (1983).

**Figure 9.**
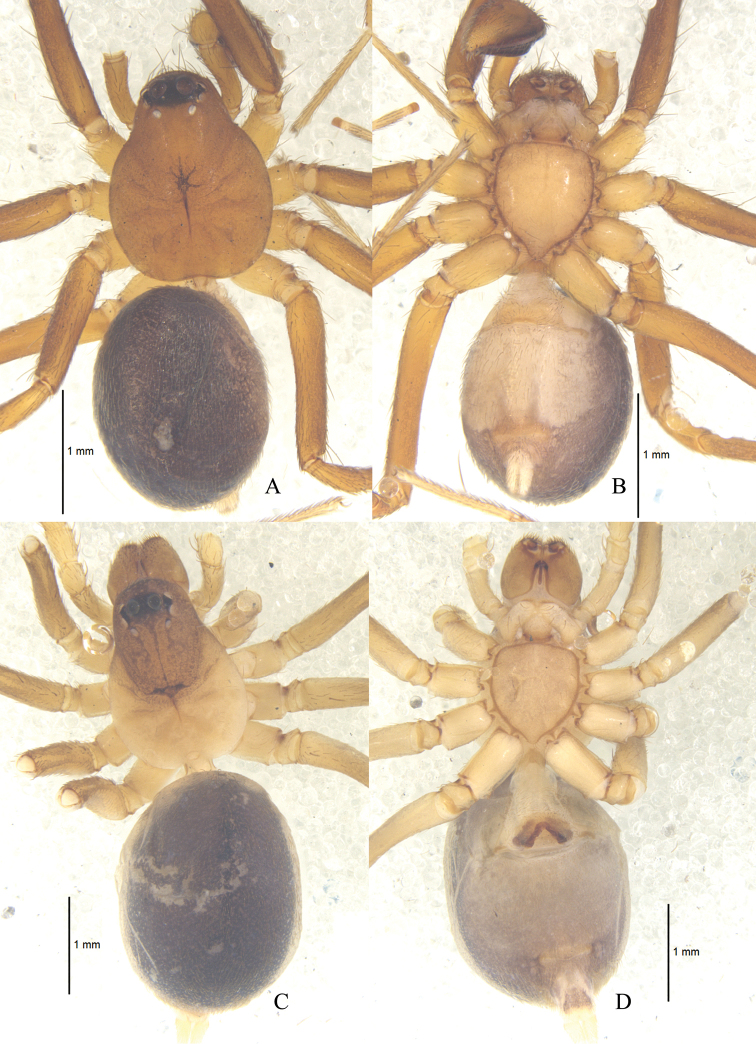
*Zodarionchaoyangense* Zhu & Zhu, 1983, male holotype (**A, B**) and female paratype (**C, D**) Habitus (**A, C** dorsal view **B, D** ventral view).

**Figure 10.**
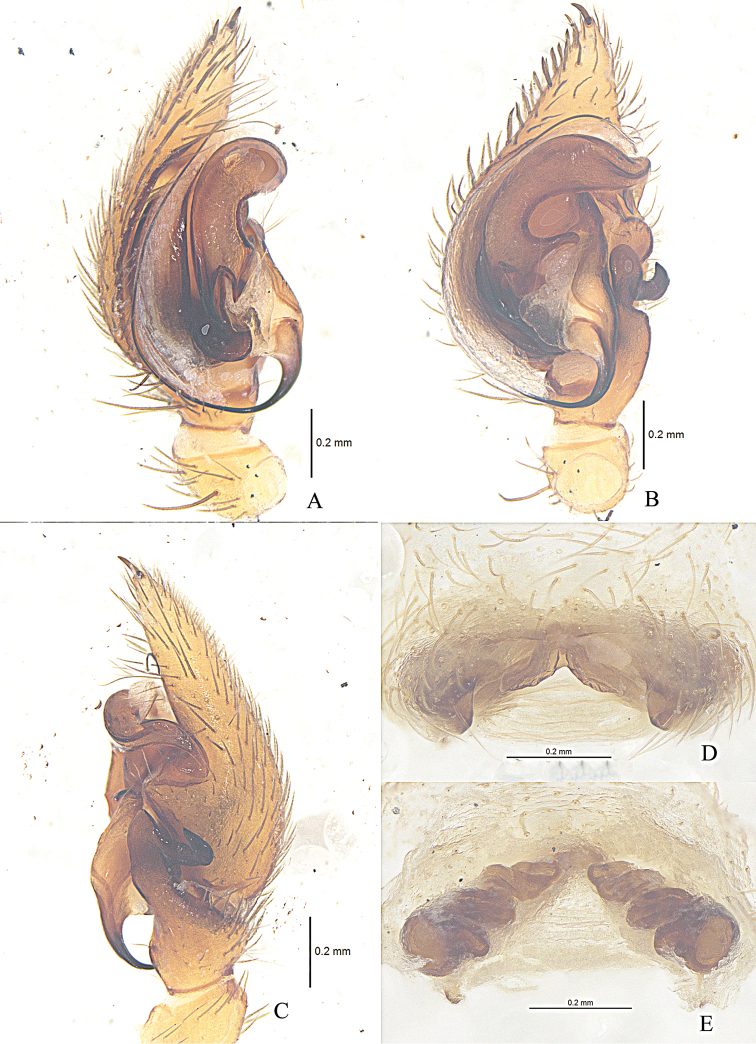
*Zodarionchaoyangense* Zhu & Zhu, 1983, male holotype left palp (**A–C**) and female paratype (**D, E**) **A** Prolateral view **B** Ventral view **C** Retrolateral view **D** Epigynum, ventral view **E** Vulva, dorsal view

##### Distribution.

China (Liaoning, Hebei).

#### Zodarion furcum

Taxon classificationAnimaliaAraneaeZodariidae

Zhu, 1988

Figures 11 , 12

Zodarion furcum Zhu 1988: 354, fig. 5–9; Song et al. 1999: 431, fig. 257R, S, 258B; Song et al. 2001: 328, fig. 211A–E; Jocqué and Henrard 2015: 21.
Zodariellum furcum Marusik and Koponen 2001: 41.

##### Material examined.

1♂ and 1♀, Shijiazhuang City (38°15'N, 114°12'E), Hebei Province, China, 17 May 1985, Mingsheng Zhu leg.

##### Description.

See Zhu (1988).

**Figure 11.**
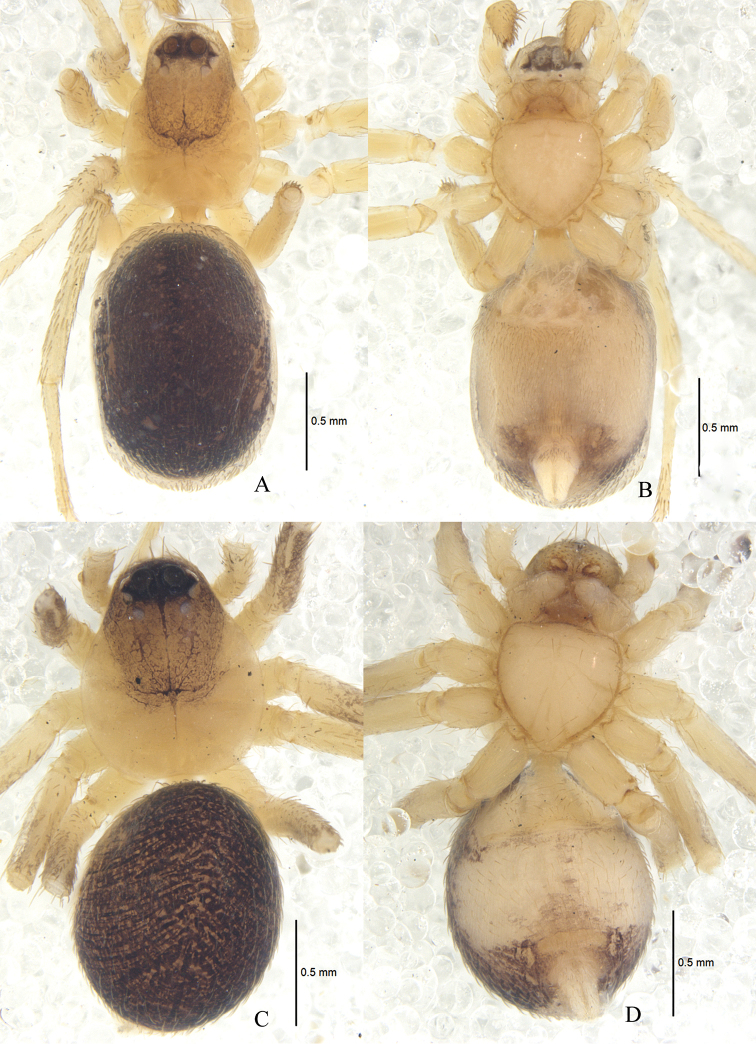
*Zodarionfurcum* Zhu, 1988, male holotype (**A, B**) and female paratype (**C, D**) Habitus (**A, C** dorsal view **B, D** ventral view).

**Figure 12.**
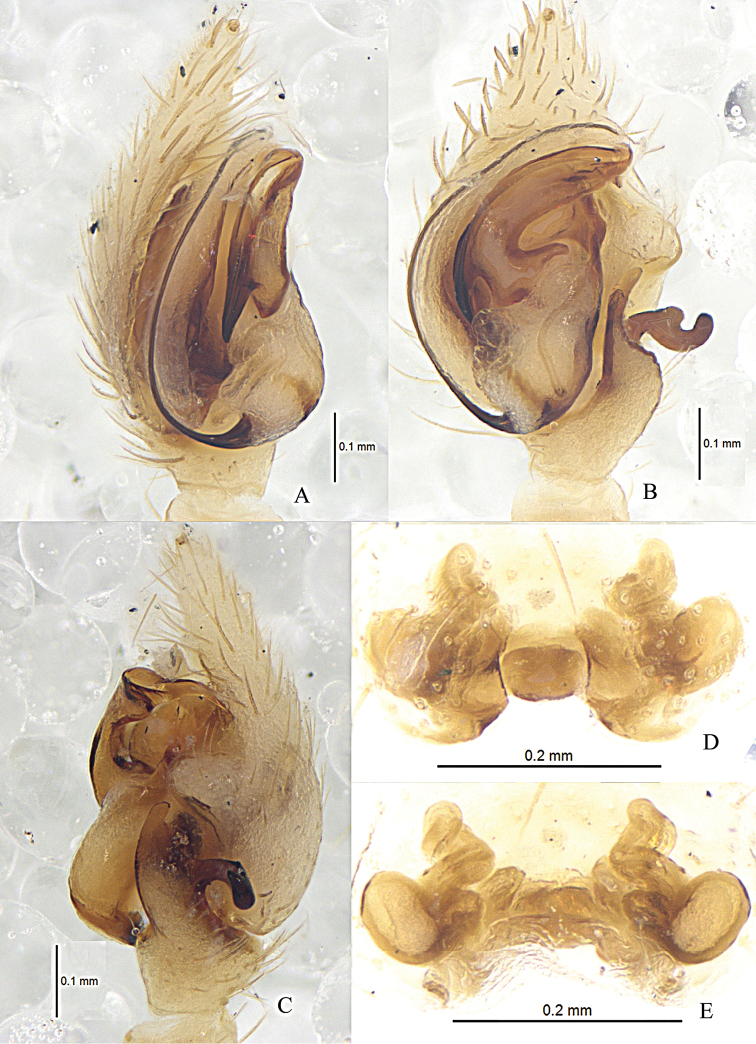
*Zodarionfurcum* Zhu, 1988, male holotype left palp (**A–C**) and female paratype (**D, E**) **A** Prolateral view **B** Ventral view **C** Retrolateral view **D** Epigynum, ventral view **E** Vulva, dorsal view.

##### Distribution.

China (Hebei).

#### Zodarion hunanense

Taxon classificationAnimaliaAraneaeZodariidae

Yin, 2012

Zodarion hunanensis Yin in Yin et al. 2012: 1148, fig. 611a–e.

##### Description and figures.

See Yin (2012).

##### Distribution.

China (Hunan).

**Figure 13.**
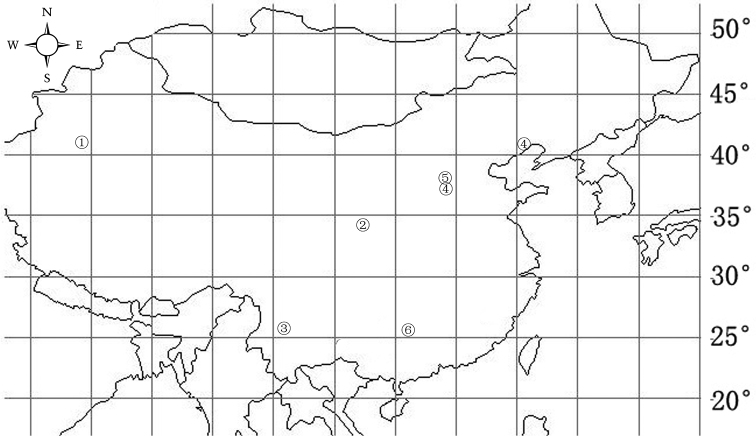
Records of *Zodarion* species in China. ① *Zodarionapertum* sp. n. ② *Zodarionplanum* sp. n. ③ *Zodarionovatum* sp. n. ④ *Zodarionchaoyangense* Zhu & Zhu, 1983 ⑤ *Zodarionfurcum* Zhu, 1988 ⑥ *Zodarionhunanense* Yin, 2012.

## Supplementary Material

XML Treatment for Zodarion

XML Treatment for Zodarion apertum

XML Treatment for Zodarion planum

XML Treatment for Zodarion ovatum

XML Treatment for Zodarion chaoyangense

XML Treatment for Zodarion furcum

XML Treatment for Zodarion hunanense

## References

[B1] Bosmans R (1994) Revision of the genus *Zodarion* Walckenaer, 1833 in the Iberian Peninsula and Balearic Islands (Araneae, Zodariidae). Eos 69: 115–142.

[B2] Bosmans R (1997) Revision of the genus *Zodarion* Walckenaer, 1833, part II. Western and Central Europe, including Italy (Araneae: Zodariidae). Bulletin of the British Arachnological Society 10(8): 265–294.

[B3] Bosmans R (2009) Revision of the genus *Zodarion* Walckenaer, 1833, part III. South East Europe and Turkey (Araneae: Zodariidae). Contributions to Natural History 12: 211–295.

[B4] Bosmans R Özkütük RS Varli SV Kunt KB (2014) Description of a new *Zodarion* Walckenaer, 1826 from Turkey (Zodariidae; Araneae). Turkish Journal of Zoology 38: 99–101. 10.3906/zoo-1303-11

[B5] Jocqué R (1991) A generic revision of the spider family Zodariidae (Araneae). Bulletin of the American Museum of Natural History 201: 1–160. http://hdl.handle.net/2246/894

[B6] Jocqué R Henrard A (2015) Revalidation of *Acanthinozodium* Denis, 1966 with description of three new species and discovery of a remarkable male palpal character (Araneae, Zodariidae). European Journal of Taxonomy 114: 1–23. 10.5852/ejt.2015.114

[B7] Li SQ Lin YC (2016) Species Catalogue of China. Vol. 2. Animals, Invertebrates (I) Arachnida: Araneae. Beijing, Science Press, 549 pp.

[B8] Marusik YM Koponen S (2001) Spiders of the family Zodariidae from Mongolia (Arachnida: Araneae). Reichenbachia 34: 39–48.

[B9] Pekár S Cardoso P Barriga JC Carvalho JC (2011) Update to the zodariid spider fauna of the Iberian peninsula and Madeira (Araneae: Zodariidae). Zootaxa 2814: 19–32. 10.11646/zootaxa.2814.1.2

[B10] Song DX Zhu MS Chen J (2001) The Fauna of Hebei, China: Araneae. Hebei University of Science and Techology Publishing House Shijiazhuang, 510 pp.

[B11] Song DX Zhu MS Chen J (1999) The Spiders of China. Hebei University of Science and Techology Publishing House Shijiazhuang, 640 pp.

[B12] World Spider Catalog (2018) World Spider Catalog, version 19.0. Natural History Museum Bern. http://wsc.nmbe.ch [Accessed on: 2018-1-17]

[B13] Yin CM Peng XJ Yan HM Bao YH Xu X Tang G Zhou QS Liu P (2012) Fauna Hunan: Araneae in Hunan, China. Hunan Science and Technology Press, Changsha, 1590 pp.

[B14] Zhu CD Zhu SF (1983) A new species of spider of the genus *Zodarium* (Araneae: Zodariidae). Journal of the Bethune Medical University 9(supplement): 137–138.

[B15] Zhu MS (1988) A new spider of the genus *Zodarium* from China (Araneae: Zodariidae). Acta Zootaxonomica Sinica 13: 353–355.

